# Is the attentional SNARC effect truly attentional? Using temporal order judgements to differentiate attention from response

**DOI:** 10.1177/17470218211039479

**Published:** 2021-08-18

**Authors:** Diana B Galarraga, Jay Pratt, Brett A Cochrane

**Affiliations:** Department of Psychology, University of Toronto, Toronto, Ontario, Canada

**Keywords:** Attention, temporal order judgement, response, SNARC

## Abstract

The spatial–numerical association of response codes (SNARC) effect reflects the phenomenon that low digits are responded to faster with the left hand and high digits with the right. Recently, a particular variant of the SNARC effect known as the attentional SNARC (which reflects that attention can be shifted in a similar manner) has had notable replicability issues. However, a potentially useful method for measuring it was revealed by Casarotti et al. using a temporal order judgement (TOJ) task. Accordingly, the present study evaluated whether Casarotti et al.’s results were reproducible by presenting a low (1) or high (9) digit prior to a TOJ task where participants had to indicate which of two peripherally presented targets appeared first (Experiment 1) or second (Experiment 2). In Experiment 1, it was revealed that the findings of Casarotti et al.’s were indeed observable upon replication. In Experiment 2, when attention and response dimensions were put in opposition, the SNARC effect corresponded to the side of response rather than attention. Taken together, the present study confirms the robustness of the attentional SNARC in TOJ tasks, but that it is not likely due to shifts in attention.

## Introduction

There is no symbolic representation more important than numbers, and accordingly, they have a profound impact on human processing. This is exemplified by the spatial–numerical association of response codes (SNARC) effect, which shows that digits influence our ability to respond (Dehaene et al., 1993; see also Wood et al., 2008). This phenomenon was first revealed using a number parity judgement task where participants had to indicate whether a centrally displayed number was odd or even. It was revealed that when low digits (i.e., 1 and 2) were presented, left-handed responses were faster than right-handed responses, and when high digits were presented (i.e., 8 and 9), right-handed responses were faster than left-handed responses. It is proposed that the SNARC effect reflects that responding to numbers is facilitated when they are spatially congruent with their arrangement on a mental number line (Dehaene et al., 1993; Gevers et al., 2006; Zorzi et al., 2002; cf. Proctor & Cho, 2006).

Some years after its discovery, the SNARC effect was expanded upon to reveal that it not only affects responding, but attentional guidance as well. In their seminal study, Fischer et al. (2003) used a cue-target procedure where a centrally displayed number (the cue) was presented to participants. Following the presentation of the cue, a target appeared in either a left or right peripheral location and participants had to detect its presence with a press of the spacebar. Conceptually identical to the response-based SNARC, it was revealed that the target was more quickly detected when it was on the left when the cue was a low digit and on the right when it was a high digit. Accordingly, it was proposed that digits too shift attention in accordance with their magnitude.

Although there have been demonstrations showing the attentional SNARC effect over the years (Dodd et al., 2008; Galfano et al., 2006; He et al., 2020; Ristic et al., 2006), there are more studies that suggest it is not observable upon replication (Bonato et al., 2008; Fattorini et al., 2015, 2016; Pellegrino et al., 2019; Pinto et al., 2018; van Dijck et al., 2014).^1^ In particular, a large-scale replication conducted by Colling et al. (2020) failed to find an attentional SNARC effect. In general, simple target detection tasks are relatively blunt instruments for detecting small RT differences, and as such, they might not be the best for assessing the attentional biases produced by digits. This leaves open the possibility that the method used to evaluate the attentional SNARC effect might be imperfectly suited for revealing it.

While RT tasks have revealed mixed results, particularly robust attentional SNARC effects were revealed by Casarotti et al. (2007) using a temporal order judgement (TOJ) task. Across six experiments, a cue-target procedure similar to Fischer et al. (2003) was used except that a target each appeared in the left and right peripheral locations. Instead of providing a response as soon as the target was detected, participants reported whether it was the left or right target that appeared first. The basis of this experimental procedure is rooted in the notion of prior entry—events that occur at attended locations are perceived sooner than those at unattended locations (Spence & Parise, 2010; Titchener, 1908). With use of a short temporal interval between targets (which made it difficult to determine which of the two targets appeared first), the researchers were able to assess whether it was the left or right target that was perceived first based on the proceeding digit cue. Using their TOJ procedure, a pattern of results was observed that was conceptually identical to the attentional SNARC effects of RT tasks; that is, participants were more likely to perceive the left target as appearing first when proceeded by a low digit and the right target as appearing first when proceeded by a high digit. Thus, across six experiments, Casarotti et al.’s TOJ procedure provides evidence in support of the attentional SNARC effect.

Given the mixed findings of the attentional SNARC effect in RTs tasks across several studies and the robustness of the findings from TOJ tasks in a single study, it seems prudent to evaluate the reproducibility of the attentional SNARC using a TOJ procedure. To accomplish this, in Experiment 1, a low digit or high digit was centrally displayed prior to a TOJ task where participants had to indicate which of two peripherally presented targets appeared first. By varying the temporal interval between targets, we were then able to calculate the point of subjective simultaneity (PSS) to evaluate whether perception varied as a function of the digit cue. If the attentional SNARC effect is indeed observable using a TOJ procedure, the PSS should be shifted to the right (left-side bias) for low digits and shifted to the left (right-side bias) for high digits. A further concern was whether an attentional SNARC effect observed using a TOJ procedure could be attributed to attentional guidance like claimed by Casarotti et al. (2007), since the left/right spatial response codes integral to the TOJ procedure may have produced this finding (see Aiello et al., 2012; Keus et al., 2005; Keus & Schwartz, 2005; Schwarz & Keus, 2004). Accordingly, in Experiment 2, participants performed a “which came second?” TOJ task (see Shore et al., 2001) where they had to report the target that appeared second by providing key-press responses on the opposite side as the perceived-first target. Given that response effects often masquerade as attentional ones (e.g., Cochrane & Milliken, 2020; Hilchey et al., 2018) orthogonalising the attention and response dimensions will allow us to determine whether the attentional SNARC in TOJ procedures was produced by shifts in attention or the left/right spatial codes for response selection.

## Experiment 1

The purpose of Experiment 1 was to evaluate whether the attentional SNARC effect was observable when digits proceeded a TOJ task (see Casarotti et al., 2007). Here, participants observed a digit cue (i.e., 1 or 9) then indicated which of two peripherally presented target circles appeared first. The temporal interval between targets varied across a set of stimulus onset asynchronies (SOAs) and from this we calculated the PSS for each digit cue (i.e., the time point at which participants’ perceived that the targets appeared simultaneous). If the attentional SNARC is observable upon replication when using a TOJ procedure, the PSS should be shifted to the right for low digits, reflecting that participants were more likely to perceive the left target as appearing first even when the right target appeared first. Conversely, the PSS should be shifted to the left for high digits, reflecting that participants were more likely to perceive the right target as appearing first even when the left target appeared first.

### Method

#### Participants

Thirty-two undergraduates from the University of Toronto participated in Experiment 1 (20 female, *M*_age_ = 19.2 years). All participants provided informed consent and had normal or corrected-to-normal visual acuity. Participants were provided either course credit or $10 CAD monetary compensation for their participation. A power analysis was conducted to establish an appropriate sample size. The effect size was computed from data of a comparable TOJ experiment conducted in the laboratory (Cohen’s *d*_z_ = 0.52). This analysis revealed that a sample size of approximately 30 participants was sufficient to assess the key effect of the experiment with power greater than .80.

#### Apparatus and stimuli

Stimuli were presented using PsychoPy v3.1.5 on a LED monitor that had a refresh rate of 144 Hz. All displays were presented on a black background with a luminance value of 0.23 cd/m^2^. All stimuli were displayed in white with a luminance value of 73.08 cd/m^2^. Each display consisted of a central fixation cross and two placeholder boxes. Each placeholder box was positioned 10° of horizontal visual angle left and right of central fixation and in the same vertical plane. The fixation cross subtended a vertical and horizontal visual angle of 0.3° and the placeholder boxes subtended a vertical and horizontal visual angle of 4°. Targets were white circles that had a diameter of 1° of visual angle and were presented centrally in the placeholder boxes. The digit cues were the numbers “1” and “9” in Helvetica font and subtended an approximate vertical and horizontal visual angle of 1°.

#### Procedure

**Figure 1.**
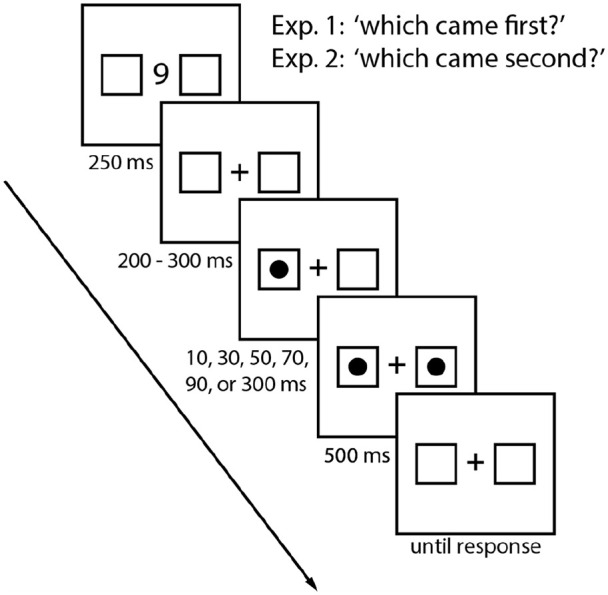
This is an example of a left-first target trial when the high digit was presented. In Experiment 1, participants were instructed to report whether the left or right target appeared first. In Experiment 2, participants were instructed to report whether the left or right target appeared second.

Participants were seated in front of a monitor and keyboard in a dimly lit room. Each trial began with a display consisting of a central fixation cross and two placeholder boxes. After 500 ms, the central fixation cross was replaced by a digit cue (i.e., 1 or 9) that was displayed for 250 ms. The digit cue displayed was randomized on a trial-by-trial basis. The digit cue was then replaced by the fixation cross for an interval that randomly fell between 200 to 300 ms. Participants then performed the TOJ task where a target circle appeared in one of the two placeholder boxes. Following one of the following SOAs: 10, 30, 50, 70, 90, or 300 ms, the second target circle appeared in the other box. Both the location of the target and the SOAs were pseudorandomized across the experimental session. Participants were required to indicate which of the target circles appeared first by pressing one of the two response keys on a standard QWERTY keyboard. To indicate that the left target appeared first they pressed the “Z” key with their left index finger and to indicate that the right target appeared first they pressed the “M” key with their right index finger. To ensure participants sufficiently attended to the digit cue, at the end of each trial they indicated the most recent digit they observed by pressing the corresponding number key.^2^ Participants initiated the start of each trial by pressing the spacebar. An example of the experimental procedure is depicted in Figure 1.

The experimental session consisted of a total of 600 trials. Given that each trial was self-paced, participants were permitted to take a break prior to each trial. Prior to the experimental session, participants performed a practice session composed of 15 trials. For the first 5 practice trials, participants performed the TOJ task without reporting the identity of the digit cue. For the next 10 practice trials, participants performed the TOJ task and reported the identity of the digit cue, like in the experimental session. The duration of the SOAs were lengthened during the practice trials to ensure that participants fully understood the task.

### Results and discussion

**Figure 2.**
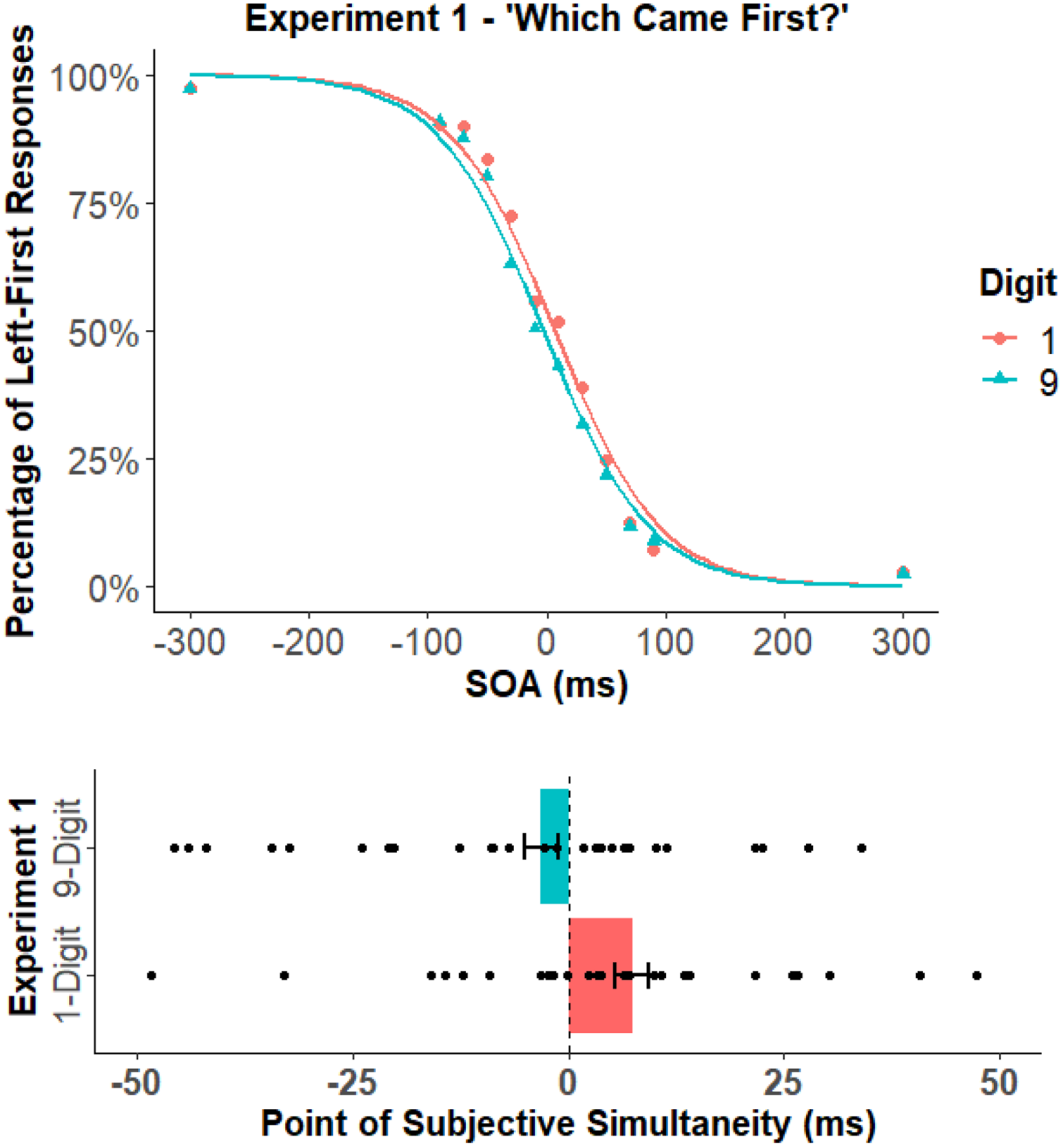
The top panel shows the mean percentage of left-first responses for the low and high digit cues of Experiment 1. The bottom panel shows the PSS values for the low and high digit cues of Experiment 1. The black circles in the bottom panel represent the PSS of individual participants. Error bars represent the standard error of the mean corrected to remove between-subject variability (Cousineau, 2005; Morey, 2008).

The primary dependent variable was the mean percentage of left-first responses. Participants with a mean percentage of left-first responses at the longest SOAs (i.e., ±300 ms) that fell outside two standard deviations of the mean of all participants at those SOAs were excluded from analysis, which led to the removal of three participants. Furthermore, all observations where participants failed to correctly identify the digit cue were removed from analysis, which led to the removal of 7.0% of observations. For the remaining observations, a logistic function was fitted to each participants’ data (i.e., the percentage of left-first responses plotted across SOAs). The PSS constituted the time point that corresponded with the point on the logistic function that indicated that left- and right-first responses were equally likely. The PSS values for each digit cue (1/9) were submitted to a two-tailed paired *t*-test. An alpha criterion of .05 was used to determine statistical significance. The complimentary Bayesian analyses accompanied all primary analyses. The models and methods of computation in all Bayesian analysis were adopted from Rouder et al. (2012). Priors for all *t*-tests were set to $\sqrt{2}/2$ to be consistent with Morey and Rouder (2011) and Rouder et al. since this value is reported to scale with effect size. The mean percentage of left-first responses and the PSS for each digit cue are depicted in Figure 2. The mean PSS and standard deviations are reported in Table 1 and the covariation matrix is reported in Table 2.

**Table 1.** The mean PSS (ms) and standard deviations (ms; in parenthesis) of Experiments 1 and 2.

	Digit	
	*1*	*9*
*Experiment 1*	7.4 (23.7)	–3.2 (25.1)
*Experiment 2*	20.7 (22.2)	26.4 (28.7)

**Table 2.** The covariance matrices of Experiments 1 and 2.

Experiment 1	*1*	*9*	Experiment 2	*1*	*9*
***1***	560.5	485.0	*1*	492.6	545.3
*9*	485.0	631.3	***9***	545.3	825.7

The PSS analysis revealed a significant effect of digit cue, *t*(28) = 3.84, *p* < .001, *d_z_* = 0.71, *BF_10_* > 30 (strong evidence supporting the H_1_).^3^ This result reflected a difference between the PSS of the 1 (*M* = 7.4 ms; *SD* = 23.7 ms) and 9 (*M* = −3.2 ms; *SD* = 25.1 ms) digit cues. In other words, the left-side target would have to lag behind the right-side target by 7.4 ms to be perceived as simultaneous when proceeded by the 1-digit cue, and the right-side target would have to lag behind the left-side target by 3.2 ms to be perceived as simultaneous when proceeded by the 9-digit cue. This finding is consistent with the conclusion that low digits shift attention to the left (thus speeding the perception of left stimuli) and high digits shift attention to the right (thus speeding the perception of right stimuli). Also, this finding demonstrates that Casarotti et al.’s (2007) TOJ results are indeed observable upon replication.^4^

## Experiment 2

While the results of Experiment 1 were consistent with the notion that the attentional SNARC effect was due to shifts of attention, it does not preclude the possibility that it may have been due to left/right response biases instead. This is because the “which came first?” TOJ task of Experiment 1 necessitated that the digit cue biased attention and response to the same side, making it impossible to tell whether it was due to shifts of attention or spatial response correspondences. In other words, this pattern of results could be produced by a response-based SNARC effect—that participants were faster to respond with their left hand for low digits and right hand for high digits (see Keus et al., 2005; Keus & Schwartz, 2005; Schwarz & Keus, 2004). To tease apart this issue, the present experiment used a procedure similar to Experiment 1 with the exception that participants made “which came second?” judgements instead. If the SNARC effect was due to shifts in attention, the pattern of results should be the same as in Experiment 1—the PSS should be shifted to the right (left-attention bias) for low digits and to the left (right-attention bias) for high digits. If the SNARC effect was due to response biases, the opposite pattern of results should be observed—the PSS should be shifted to the left (right-hand bias) for low digits and to the right (left-hand bias) for high digits.

### Method

#### Participants

Thirty-seven undergraduates from the University of Toronto participated in Experiment 2 (28 female, *M*_age_ = 18.7 years). All participants provided informed consent and reported normal or corrected-to-normal visual acuity. Participants were provided either course credit or $10 CAD monetary compensation for their participation. The sample size was selected to be similar to Experiment 1.

#### Apparatus and stimuli

The apparatus and stimuli were identical to Experiment 1.

#### Procedure

The procedure and design were identical to Experiment 1 with the exception that participants indicated which of the two peripherally presented target circles appeared second.

### Results and discussion

**Figure 3.**
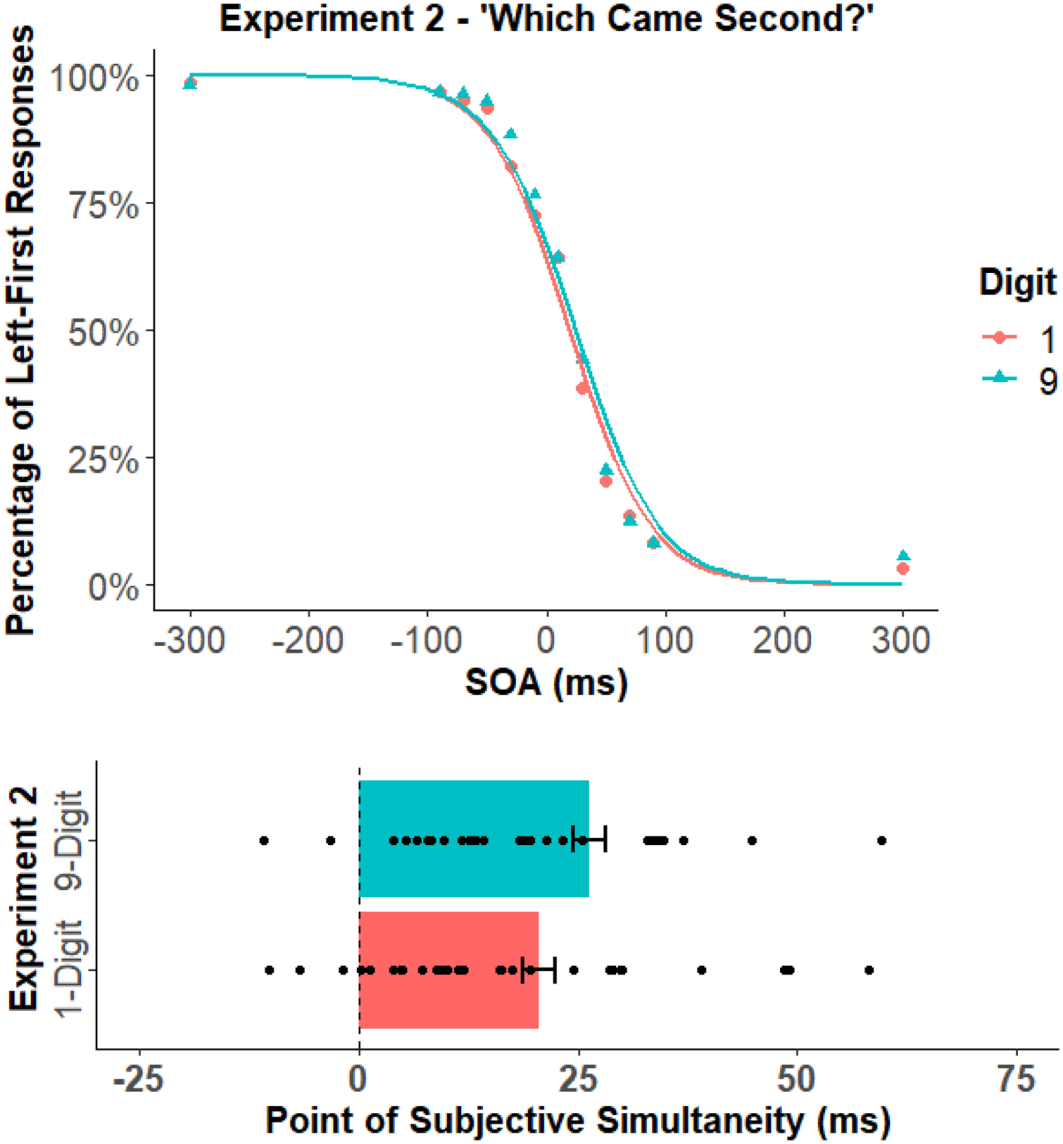
The top panel shows the mean percentage of left-first responses for the low and high digit cues of Experiment 2. The bottom panel shows the PSS values for the low and high digit cues of Experiment 2. The black circles in the bottom panel represent the PSS of individual participants. Error bars represent the standard error of the mean corrected to remove between-subject variability (Cousineau, 2005; Morey, 2008).

The primary dependent variable was the percentage of left-first responses. Participants with a mean percentage of left-first responses at the longest SOAs (i.e., ± 300 ms) that fell outside two standard deviations of the mean of all participants at those SOAs were excluded from analysis, which led to the removal of four participants. All observations where participants failed to correctly identify the digit cue were removed from analysis, which led to the removal of 8.4% of observations. The PSS was computed from the remaining observations by fitting a logistic function to each participants’ data. The PSS values for each digit cue (1/9) were then submitted to a two-tailed paired *t*-test. An alpha criterion of .05 was used to determine statistical significance. The complementary Bayesian analyses accompanied all primary analyses. As before, the models and methods of computation in all Bayesian analysis were adopted from Rouder et al. (2012). Priors for all *t*-tests were set to $\sqrt{2}/2$ to be consistent with Morey and Rouder (2011) and Rouder et al. The mean percentage of left-first responses and the PSS for each digit cue are depicted in Figure 3. The mean PSS and standard deviations are reported in Table 1 and the covariation matrix is reported in Table 2.

The PSS analysis revealed a significant effect of digit cue, *t*(32) = 2.22, *p* = .034, *d_z_* = 0.39, *BF_10_* = 1.59 (weak evidence supporting the H_1_).^5^ This result reflected a difference between the PSS of the 1 (*M* = 20.7 ms; *SD* = 22.2 ms) and 9 (*M* = 26.4 ms; *SD* = 28.7 ms) digit cues. Importantly, this pattern of results was reversed relative to Experiment 1; the PSS of the 9-digit cue was greater than the 1-digit cue in the present experiment, whereas the PSS of the 1-digit cue was greater than the 9-digit cue in Experiment 1. In other words, the bias produced by the digit cue corresponded with the response side rather than the side of attention. This finding supports the notion that the attentional SNARC effect was due to response biases rather than shifts of attention.^6^

### Experiment 1 and 2 comparison

We conducted a mixed factor ANOVA that treated TOJ task (‘which came first?’/’which came second?’) as a between-subjects factor and digit cue (1/9) as a within-subject factor. Once again, the models and methods of computation of the complimentary Bayesian analysis were adopted from Rouder et al. (2012). As advocated by Rouder et al., priors for fixed effects were set to 0.5 and random effects were set to 1. This analysis revealed a significant interaction of TOJ task and digit cue, *F*(1, 60) = 18.6, *p* < .001, $\eta_{p}^{2}=.24$ , *BF_10_* > 30 (strong evidence supporting the H_1_), which further supports that the digit cue biased the side of response rather than attention. This analysis also revealed a significant main effect of TOJ task, *F*(1, 60) = 12.3, *p* < .001, $\eta_{p}^{2}=.17$ , *BF_10_* = 29.8 (strong evidence supporting the H_1_), reflecting an overall greater left-side bias for the participants that performed the “which came second?” than the “which came first?” TOJ task.

## General discussion

The purpose of the present study was to evaluate whether the attentional SNARC effect was robust while using a TOJ procedure like in Casarotti et al. (2007). To do so, a high or low digit was presented prior to a TOJ task where participants had to indicate which of two peripherally presented target circles appeared first (Experiment 1) or second (Experiment 2). The results of Experiment 1 revealed that the findings of Casarotti et al. (2007) were indeed observable upon replication—that participants were more likely to report that the left target appeared first following a low digit and that the right target appeared first following a high digit. In Experiment 2, when attention and response demands were put in opposition, the pattern of results significantly reversed such that the SNARC effect corresponded to the side of response—that participants were more likely to report that the right target appeared first (left-side response) following a low digit and that the left target appeared first (right-side response) following a high digit. In other words, while the results of Casarotti et al. were indeed reproducible, they did not appear to be due to shifts in attention.

While the present findings suggest that the attentional SNARC effect is not due to attention, it is possible that the constituent data pattern can be produced by other (non-attentional) processes. One set of processes are those underlying the manual response behaviours thought to constitute the classic SNARC effect. That is, the congruency of number magnitude and the side of space can lead to efficient responding when left-handed responses are made to low digits and right-handed responses are made to high digits (Keus et al., 2005; Keus & Schwartz, 2005; Schwarz & Keus, 2004). While many studies investigating the attentional SNARC use detection tasks (which should control for the spatiality of manual responses), elimination from the contribution of these response processes depends on participants not varying their response hand. If participants changed their response hand based on the digit cue, this response selection behaviour would produce a Simon effect (Hedge & Marsh, 1975; Simon, 1969; Simon & Rudell, 1967). Given that participants will respond to low digits with their left hand and high digits with their right when given the choice (Daar & Pratt, 2008), left-handed responses should be faster than right-handed responses for left-sided targets and vice versa; a result that could explain the attentional SNARC finding. Another set of processes that can produce the attentional SNARC are those involved with actively contemplating the digit cue position on a mental number line. That is, it has been demonstrated that the attentional SNARC effect appears to be robust when participants detect the target onset by saying aloud whether the digit cue fell left or right of five on a mental number line (Fattorini et al., 2015, 2016; Pinto et al., 2018). This is to suggest that the attentional SNARC effect does not emerge automatically, but it can when the task is to consider the digit’s correspondence on a mental number line.

It is worth noting that, at first glance, the interpretations espoused in the present manuscript are incompatible with the findings of Casarotti et al.’s (2007) sixth experiment. In this experiment, a TOJ procedure was used like that here except that participants provided arbitrary vocal responses to indicate the side of the first target (i.e., “fulpo” to indicate left and “pingo” to indicate right). This was done to remove any biases produced by the manual response procedure used in their previous five experiments. It was revealed that with vocal responses, the attentional SNARC effect was observable. While these findings cannot be accounted for by manual response behaviour specifically, the arbitrary vocal responses were still associated with left and right. That is, while the SNARC effect can be produced by left/right manual response demands (Fias, 2001; Fias et al., 1996; Keus et al., 2005; Keus & Schwartz, 2005 ; Schwarz & Keus, 2004), it can also be produced when spatial codes are linked to numerical features in the absence of them (Fischer & Shaki, 2017, 2018; Pinto et al., 2019, 2021) This is to indicate that their vocal response procedure does not necessarily indicate that their attentional SNARC finding reflected shifts in attention.

An unexpected finding in the present study was that the “which came second?” TOJ task led to a greater proportion of overall left-first responses relative to the “which came first?” TOJ task, which led to a similar proportion of left- and right-first responses. It is important to note the difference across these experiments was purely instructional—the program code and other methodological aspects of the experimental design were identical. Furthermore, this finding was not due to a few outlier participants as all but two participants showed an overall left-first bias (see Figure 3). Given that we suspect this result is not spurious, it begs the question: what is it about “which came second?” judgements that caused a left-side bias? We suspect it was caused by task difficulty. When participants performed the “which came first?” judgement, they could use the automatic capture of the abrupt onset to guide their response (see Remington et al., 1992; Yantis & Jonides, 1990). In contrast, when participants performed the “which came second?” judgement, the incongruency of abrupt onsets and response side made it a more confusing task. This confusion then caused participants to rely on well-learned behaviours; in particular, the biasing of left-side visual information that is prevalent in humans (Gilbert & Bakan, 1973; Vaid & Singh, 1989). Overall, while we suspect that uncertainty increased reliance on well-learned behaviours, more research is needed to validate our supposition.

Another interesting aspect of the present study is that the effect size was larger in Experiment 1 (*d_z_* = 0.71) than in Experiment 2 (*d_z_* = 0.39). We suspect the reason for the relatively tenuous effects in Experiment 2 was that the results did not purely reflect the contribution from a response process. As noted above, the SNARC effect can be produced by the spatial dependencies of the task independent of left/right manual response demands. For example, items maintained in working memory can bias responding in accord with their spatial correspondence (Abrahamse et al., 2016; Fias et al., 2011; Fias & van Dijck, 2016; van Dijck et al., 2013, 2014; van Dijck & Fias, 2011) such that left/right spatial correspondences in working memory produce biases like those of left/right responses in space. Accordingly, it is possible that the findings of Experiment 2 reflected a SNARC effect produced by the biasing of manual response behaviour, but it was somewhat disrupted by the spatial correspondence of the “which came second?” instruction. In addition, it is highly plausible that the overall left-side bias produced by the “which came second?” judgement made the effect of digit cue less observable.

One thing to keep in mind was that participants in the present study had to report the identity of the high and low digit following each trial of the TOJ task by pressing the corresponding number key on a standard QWERTY keyboard. This digit task was based on the findings of Zanolie and Pecher (2014), which showed that attentional SNARC effects were only present when participants actively processed the digit. The decision to include this digit task in the present TOJ procedure was made because we deemed it inappropriate to challenge the reproducibility of Casarotti et al. (2007)’s findings with only a weak attempt to find them. However, it is possible that this digit task influenced the TOJ results given the left-to-right organisation of these digits on the keyboard. While it is unclear how the digit task could explain the opposite effects of Experiments 1 and 2, there may be reason to suspect it influenced their magnitude. That is, in addition to the increased complexity of the “which came second?” instruction as noted above, it could be that the left/right incongruency of the TOJ and digit task responses in Experiment 2 attenuated the effect. Or that the left/right congruency of the TOJ and digit task responses of Experiment 1 enlarged the effect. Overall, while the digit task served some benefit as it permitted the removal of observations when participants failed to process the digit cue, it is unclear whether it may have influenced the effects of the TOJ task.

## Conclusion

The present study demonstrates that an attentional SNARC effect can be found with temporal order judgements but is not likely due to shifts in attention. Using a “which came second?” TOJ task that orthogonalized the attention and response dimensions, we observed a pattern of results that was opposite to that of the attentional SNARC. Together these experiments support the notion that irrelevant low and high digits can bias the side of response in TOJ tasks.

## Notes

1. It is worth noting that, while the data pattern constituting the attentional SNARC was revealed by Galfano et al. (2006) and Ristic et al. (2006), their conclusions differ from Fischer et al. (2003); they propose that the attentional SNARC is not automatic, as it is modulated by top-down factors.

2. The decision to have participants report the digit cue following each trial was based on the results of Zanolie and Pecher (2014), which indicated that the attentional SNARC may depend on the digit cue being processed.

3. The same PSS analysis was conducted without excluding observations when participants failed to correctly identify the digit cue. This analysis revealed a significant effect of digit cue, *t*(28) = 2.82, *p* = .009, *d_z_* = 0.52, *BF_10_* = 5.07 (moderate evidence supporting the H_1_), reflecting a different PSS produced by the 1 (*M* = 4.6 ms; *SD* = 24.1 ms) and 9 (*M* = −2.5 ms; *SD* = 25.2 ms) digit cues.

4. A one sample *t*-test was conducted when collapsing across the levels of digit cue to assess whether digits produced a bias that differed from zero. This analysis did not reveal a significant effect, *t*(28) = 0.50, *p* = .62, *d_z_* = 0.09, *BF_10_* = .22 (moderate evidence supporting the H_0_), indicating that the PSS (*M* = 2.1 ms; *SD* = 23.2 ms) did not differ from zero.

5. The same PSS analysis was conducted without excluding observations when participants failed to correctly identify the digit cue. This analysis revealed a marginally significant effect of digit cue, *t*(32) = 1.98, *p* = .057, *d_z_* = 0.34, *BF_10_* = 1.04 (weak evidence supporting the H_1_), reflecting a different PSS produced by the 1 (*M* = 22.2 ms; *SD* = 22.8 ms) and 9 (*M* = 27.6 ms; *SD* = 30.9 ms) digit cues.

6. A one sample *t*-test was conducted when collapsing across the levels of digit cue to assess whether digits produced a bias that differed from zero. This analysis revealed a significant effect, *t*(32) = 5.50, *p* < .001, *d_z_* = 0.96, *BF_10_* > 30 (strong evidence supporting the H_1_), indicating that the PSS (*M* = 23.5 ms; *SD* = 24.5 ms) was biased to the left side.
